# Emerging Zn Anode‐Based Electrochromic Devices

**DOI:** 10.1002/smsc.202100040

**Published:** 2021-09-21

**Authors:** Wu Zhang, Haizeng Li, William W. Yu, Abdulhakem Y. Elezzabi

**Affiliations:** ^1^ Institute of Frontier & Interdisciplinary Science Shandong University Qingdao 266237 China; ^2^ Ultrafast Optics and Nanophotonics Laboratory Department of Electrical and Computer Engineering University of Alberta Edmonton Alberta T6G 2V4 Canada

**Keywords:** electrochromic displays, electrochromism, smart windows, Zn anode-based electrochromic devices

## Abstract

The development of electrochromic materials has opened the door to the development of numerous devices including smart windows, color displays, optical filters, wearable camouflages, among others. Although the current electrochromic devices do not consume energy while maintaining their colored or colorless states, their bistable operation requires external electrical energy to be consumed during switching. To reduce the energy consumption of an electrochromic device, an emerging Zn anode‐based electrochromic device concept was recently introduced to partially retrieve the consumed electrical energy. In this Review, key technological developments and scientific challenges are presented for a broad range of Zn anode‐based electrochromic device configurations with emphasis on the inherent distinctions between the Zn anode‐based and conventional electrochromic devices. Specifically, a comprehensive comparison of the two classes of electrochromic devices in the high‐performance device design is provided. For the electrochromic layer, the methods for obtaining high‐quality electrochromic materials and thin films are reviewed. For the electrolytes, the effect of the dual ion system on the electrochromic performance is discussed. Also, some critical but unresolved issues in the device design and fabrication are discussed. The perspectives and outlook at the end of this Review provide recommendations to improve performance for future electrochromic studies.

## Graphical abstract

Zn anode‐based electrochromic device technology provides a new strategy to save the electrical energy consumed during color switching, where the self‐coloration behavior and energy retrieval functionality can render highly energy‐efficient devices. Among the numerous electrochromic devices developed, this compelling device emerges to be one of the most advanced and promising electrochromic technology platforms.

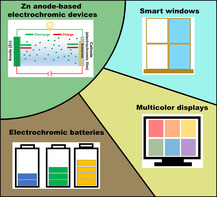

## 1 Introduction

Electrochromism is an intriguing phenomenon by which electrochemical oxidation and reduction induce a reversible color change in material.^[^ 1, 2 ^]^ This compelling light control technology endows a variety of applications, including electronic skins,^[^ 3, 4 ^]^ displays,^[^ 5, 6, 7, 8 ^]^ autodimming rearview mirrors,^[^ ^9^ ^]^ and smart windows.^[^ 10, 11, 12, 13 ^]^ Such platforms can retain their colored or bleached states in an open‐circuit configuration (i.e., zero energy consumption) for most inorganic electrochromic materials or some modified organic electrochromic materials,^[^ 14, 15, 16, 17, 18 ^]^ requiring only the application of a small external voltage to trigger the bistable coloration and decoloration states. Compared with self‐bleaching materials,^[^ 19, 20 ^]^ this characteristic bistability offers a great advantage for energy‐efficient applications. As such, the bistability of organic electrochromic materials can be significantly enhanced via various approaches. For example, Wang et al. developed an ideal bistable electrochromic display based on concerted intramolecular proton‐coupled electron transfer.^[^ ^16^ ^]^ Shin et al. significantly improved the bistability of conjugated polymers by designing an electrochemical double layer.^[^ ^18^ ^]^ Along with light control, an interesting feature of electrochromic devices is their supercapacitive ability to store and release a significant amount of the consumed electrical energy.^[^ 21, 22, 23 ^]^ Notwithstanding, the zero energy consumption advantage results in a slow and complex energy release mechanism. Therefore, to meet the fast switching requirement, an external voltage bias is still required to reverse the colored state of the device.^[^ 23, 24, 25, 26, 27 ^]^

Although the conventional electrochromic devices can also be used to store energy, the operation of conventional electrochromic devices requires external voltages to trigger both the coloration/bleaching processes,^[^ 13, 14 ^]^ which makes the conventional electrochromic devices far from a net‐zero energy‐consumption technology. Nevertheless, the majority of the current electrochromic device research has focused on developing electrochromic materials for fast response without paying attention to reducing the consumed energy of the electrochromic devices. Recently, we developed a promising Zn‐based electrochromic device (ZECD) platform.^[^ 28, 29, 30 ^]^ The ZECD platform not only reduces the energy consumption during operation, via the energy retrieval functionality, but also exhibits a rapid spontaneous switching behavior due to the high value of redox potential difference between the metal anode and the electrochromic cathode. ZECDs that incorporate electrochromic phenomenon and energy storage functionalities in a single platform are examples of innovative technologies with great potential. Moreover, the aqueous compatible Zn anode implements a much lower charging voltage for the electrochromic battery when compared with the Li^+^ and Al^3+^‐based electrochromic batteries.^[^ 31, 32, 33 ^]^ The lower charging voltage indicates a lower energy consumption during the bleaching process. As such, the Zn‐based aqueous electrochromic battery platform emerged to be one of the most advanced and promising electrochromic technology platforms.

With recent the rapid development of electrochromic technology, there is an urgent requirement for high efficiency and ultralow energy consumption electrochromic devices for various applications.^[^ ^34^ ^]^ Comprehensive reviews on conventional electrochromic devices outlining their respective strengths and limitations have served to address some of such challenges.^[^ 34, 35, 36, 37, 38, 39, 40, 41, 42, 43, 44, 45, 46 ^]^ In light of these investigations, we offer a critical comprehensive review focusing on the emerging ZECDs and their applications. This Review serves to complement the existing review articles in this field and aims to discuss the distinctive aspects of the newly born ZECDs in comparison with other well‐established electrochromic devices. Herein, the basic mechanisms, device structures and electrochemical processes involved in the ZECDs and conventional electrochromic devices are introduced, followed by discussions on the major advancements, future challenges, and finally, presenting new opportunities in ZECD technology and point out the future directions of related research.

## 2 ZECDs versus Conventional Electrochromic Devices

### 2.1 Basic Principles

**Figure 1:**
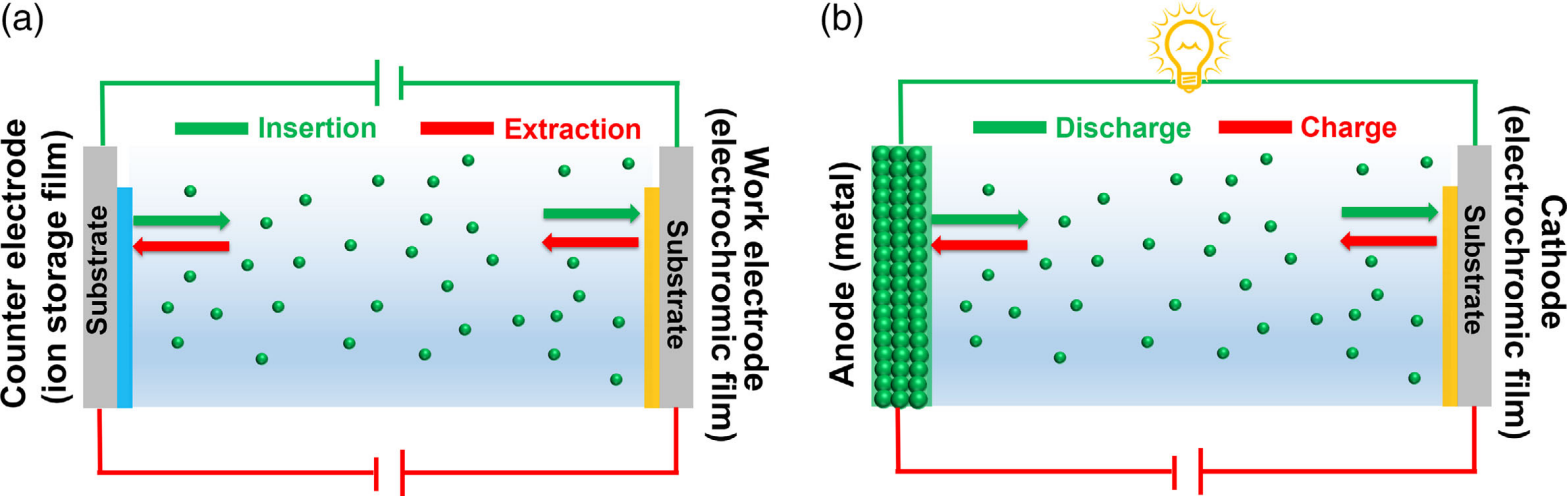
Comparison between a) conventional electrochromic supercapacitor and b) electrochromic battery.

Conventional electrochromic devices are composed of an electrochromic layer, a counter layer, an electrolyte layer, and two transparent conducting substrates (**Figure** **1**a).^[^ ^47^ ^]^ Upon the application of a potential difference between the conducting substrates, such an electrochromic device changes its optical properties due to the injection of charge into the electrochromic layer. This charge injection is simultaneously followed by the movement of counter ions or electrons to balance the charge in the counter layer. Typically, these devices comprise two matched coloring layers; an anodic layer for anodic coloration and a cathodic layer for cathodic coloration.

In general, the optical contrast of a device mainly depends on the thickness of the electrochromic layer.^[^ ^48^ ^]^ As the film thickness increasing, the transmittance of both the colored and bleached states decreases. Typically, the maximum optical contrast is reported at an attained intermediate thickness of a few hundred nanometers.^[^ 49, 50 ^]^ Within the electrochromic research community, it has been widely accepted that electrochromic devices are essentially thin‐film electrical supercapacitors and as a result, they possess low capacitance.^[^ 34, 51, 52 ^]^ However, as the electrochromic film's reversible color change is associated with the faradic reactions (i.e., ion intercalation/deintercalation), the electrochromic device behavior is similar to the pseudocapacitor.^[^ ^53^ ^]^ For example, if the electrochromic film shown in Figure 1a is composed of a cathodic electrochromic material, the colored electrochromic device can be considered as a charged pseudocapacitor.^[^ ^23^ ^]^ During the charging process, the external voltage acts as the driving force to trigger the guest ion intercalation into the electrochromic layer, thus, resulting in a corresponding coloration. Conversely, during the discharging process, guest ions are partially released from the electrochromic materials and intercalated into the counter layer. In this regard, the colored electrochromic device can be utilized to supply electrical energy, hence, recycling the energy consumed during the coloring process of the device. However, as for the electrochromic materials where light modulation is triggered by guest ions, strong electrostatic interactions are formed between embedded ions and electrochromic films. As such, the high bistability implies that the guest ions cannot be spontaneously and completely extracted from the colored electrochromic film.^[^ 13, 30 ^]^ This inherent characteristic indicates that the electrical energy retrieved from that consumed during the coloring process is very limited. Most importantly, the colored electrochromic device cannot be fully bleached via draining its electrical power in an external load (i.e., via powering external electronic devices). An external bias is still required to bleach the electrochromic device due to the fast switching requirement.^[^ 21, 22, 24 ^]^

In contrast, in an electrochromic battery configuration (Figure 1b), a metal (e.g., Al, Zn) serves as an anode, whereas an electrochromic film serves as a cathode. When the two electrodes are connected together, the redox potential difference between anode and cathode acts as a driving force to cause the metal (anode) to be oxidized and the electrochromic material to be reduced.^[^ 29, 54 ^]^ The electrochromic material acquires these electrons and metal ions for the spontaneous color change process. Interestingly, the spontaneous color switching, which is a Gibbs free energy downhill process, is the same as the discharge process in a battery. Conversely, during the oxidation process (the reverse color switching), metal ions are extracted from the reduced electrochromic cathode and metal atoms are plated onto the anode. This process is also the same as the one taking during the charging process in a half‐cell battery.^[^ ^55^ ^]^ In essence, this electrochromic battery is different from those conventional electrochromic energy storage devices as it eliminates the need for an external bias needed to reduce the electrochromic cathode. Furthermore, this platform provides an excelling function to retrieve the consumed energy during the oxidation process. The electrochromic battery device platform can be regarded as the “inverse device” of conventional electrochromic technology. Such emerging technology is highly attractive for the development of next‐generation electrochromic devices. In the following section, we highlight the research progress of conventional electrochromic technology and the newly emerging electrochromic battery technology. By introducing various materials, nanostructured interfaces, nanofabrication approaches, and device schemes, we aim to provide an overall summary and point out the future directions of related research.

### 2.2 Anodes and Cathodes

#### 2.2.1 Complementary Electrochromic Supercapacitors

Electrochromic materials are the key component in the electrochromic devices to realize reversible color switching. Considering the operating principle difference of electrochromic materials, they can be classified into two types: “cathodic electrochromic materials”, which tints its color under ion insertion, and “anodic electrochromic materials” which tints its color under ion extraction. The optimal design of a complementary electrochromic supercapacitor is to employ a cathodic electrochromic material as the electrochromic layer and incorporate an anodic electrochromic material as the counter layer.^[^ 56, 57, 58 ^]^

Ideally, when these two types of electrochromic materials having matched coloring layers are configured into a single electrochromic supercapacitor, they function to result in a more efficient and visually pleasing light‐controlled device.^[^ ^59^ ^]^

##### Cathodic Electrochromic Materials

**Figure 2:**
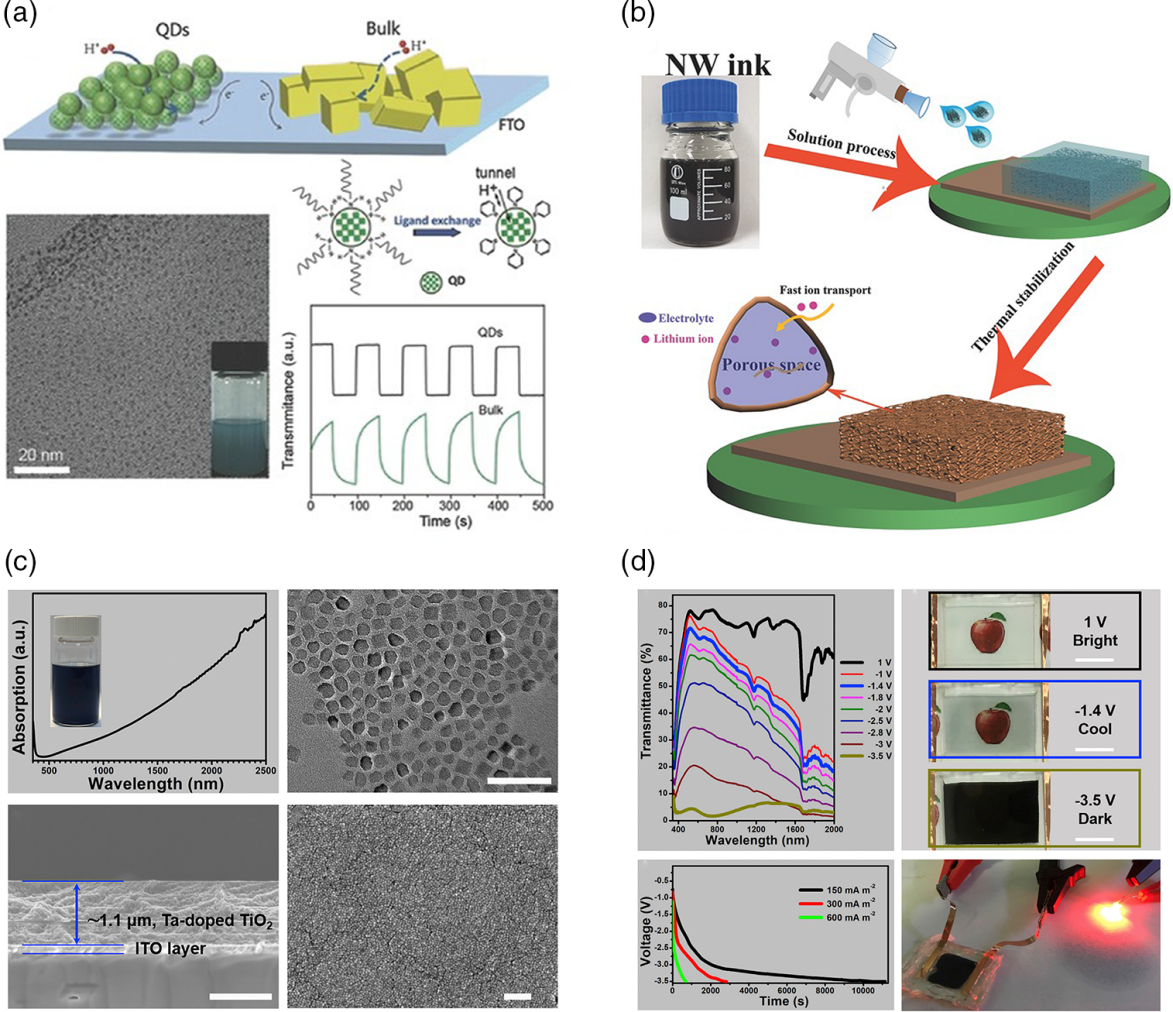
a) WO_3–*x*_ quantum dots for fast electrochromic supercapacitors. Reproduced with permission.^[^ ^60^ ^]^ Copyright 2014, Wiley‐VCH. b) Schematic illustration of the spray‐coating process for fabricating a porous Mo‐doped WO_3_ NW electrochromic supercapacitor electrode. Reproduced with permission.^[^ ^21^ ^]^ Copyright 2017, Wiley‐VCH. c) The spin‐coated Ta‐doped TiO_2_ films. d) The Ta‐doped TiO_2_ films for electrochromic supercapacitors. c,d) Reproduced with permission.^[^ ^23^ ^]^ Copyright 2019, Elsevier.

In an electrochromic supercapacitor, the most commonly used cathodic electrochromic material is WO_3_. Cong et al. fabricated single‐crystalline WO_3_ quantum dots for fast electrochromic supercapacitor applications.^[^ ^60^ ^]^ The WO_3_ quantum dots were shown to greatly shortens the diffusion paths of intercalation ions in the solid phase, thus realizing fast charge transfer and color switching (**Figure** **2**a).^[^ ^61^ ^]^ With an average size down to 1.6 nm, the WO_3_ quantum dots exhibited coloration/bleaching times within 1 s. Doping technique is considered to be an alternative method to reduce the size of the WO_3_ nanomaterials.^[^ ^62^ ^]^ Li et al. synthesized high‐quality aqueous nanowire (NW) ink via doping molybdenum in WO_3_. The aqueous NW ink was compatible with a versatile spray‐coating method for fabricating porous electrochromic supercapacitor electrodes (Figure 2b).^[^ ^21^ ^]^ It was observed that the porous structure favorably enhances the ion transport kinetics through the film.^[^ ^22^ ^]^ In addition to WO_3_, TiO_2_ is another common cathodic electrochromic material for electrochromic supercapacitors. Tong et al. used a hard template method to prepare TiO_2_ mesoporous nanotube array electrodes.^[^ ^63^ ^]^ The TiO_2_ nanotube array electrodes exhibited strong electrochromic contrast and a high‐rate capability in the fast galvanostatic charge/discharge process.^[^ ^63^ ^]^ For example, the TiO_2_ nanotube array electrodes delivered a high specific capacity of 60 mAh g^−1^ at 1 A g^−1^, accompanied by an optical contrast of 30.4% at 700 nm. Cao et al. reported that the Ta^5+^ substitution of Ti^4+^ cations generates free carriers in the TiO_2_ conduction band and result in strong localized surface plasmon resonance absorption in the near‐infrared region.^[^ ^64^ ^]^ Ta‐doped TiO_2_ nanocrystals showed great promise for visible light and near‐infrared dual‐band electrochromic supercapacitor applications (Figure 2c,d).^[^ ^23^ ^]^ Excellent electrochromic performance in terms of an impressive light modulation for visible and near‐infrared regions (89.1% at 550 nm and 81.4% at 1600 nm) and good electrochemical stability (the optical modulation at 550 and 1600 nm decreased by 0.2% and 6.0%, respectively, after 2000 cycles) were demonstrated. Moreover, the Li_4_Ti_5_O_12_ (LTO) electrode also exhibited good electrochromic performance in terms of visible and near‐infrared light modulation (54.9% at 550 nm and 71.6% at 1000 nm).^[^ ^65^ ^]^ When assembled with a complementary NiO electrode, the rechargeable electrochromic device showed a high operating voltage of 3.0 V which could power a 1.7 V red light‐emitting diode (LED) for more than 10 min and provided an energy density of 0.2 Wh cm^−3^.

**Figure 3:**
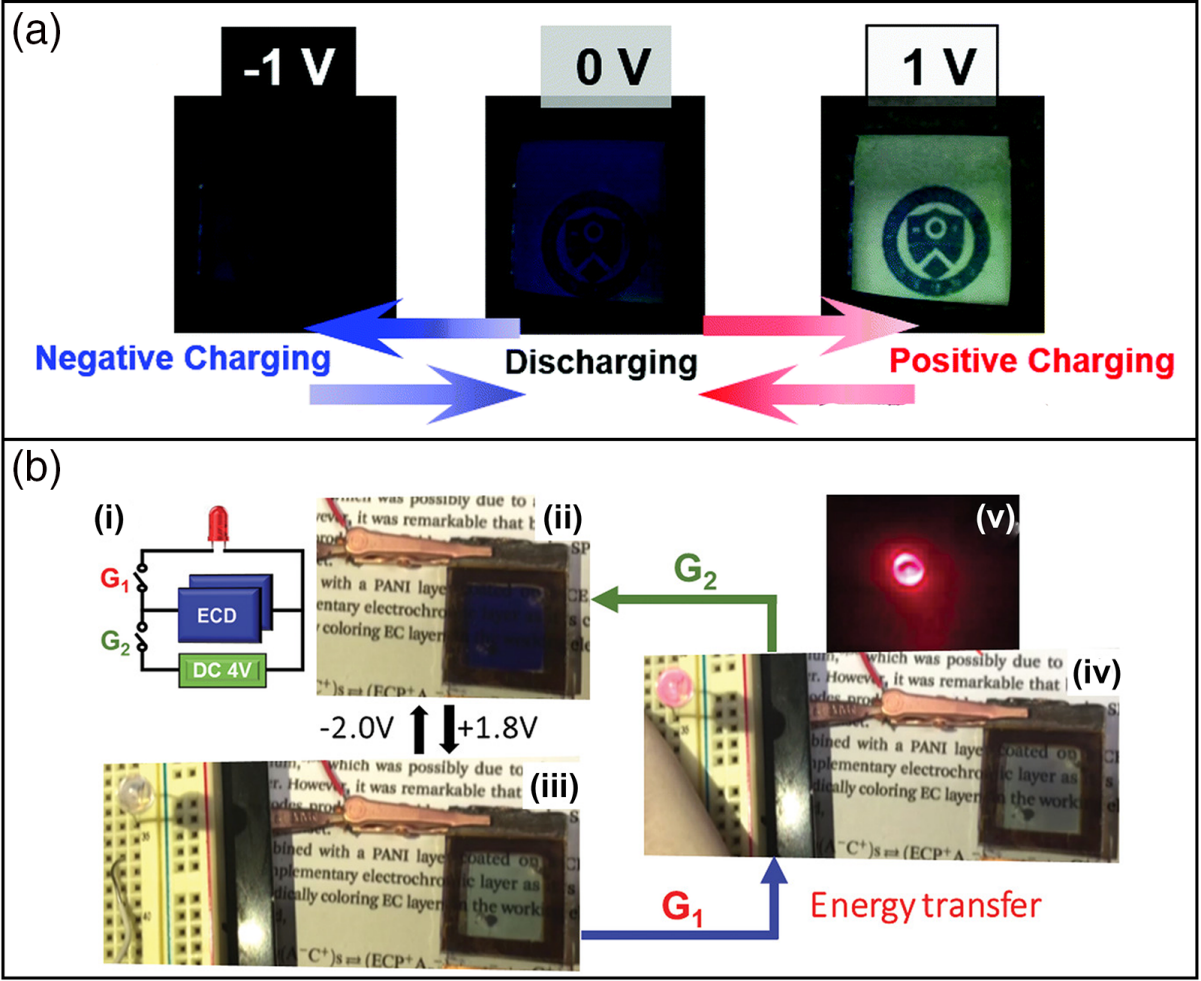
a) Images of the PR–Br‐based electrochromic supercapacitor at different energy states. Reproduced with permission.^[^ ^25^ ^]^ Copyright 2018, Royal Society of Chemistry. b) The energy transfer functionality of the PR–Br‐based electrochromic supercapacitors. Reproduced with permission.^[^ ^26^ ^]^ Copyright 2019, Wiley‐VCH.

Cathodic conjugated electrochromic polymers have also shown potential for electrochromic supercapacitor applications.^[^ ^66^ ^]^ Li et al. reported on titanium carbide‐poly(3,4‐ethylenedioxythiophene) (PEDOT) for electrochromic microsupercapacitors use.^[^ ^67^ ^]^ The PEDOT endowed an electrochromic effect for MXene‐based microsupercapacitors with switching times of 6.4 and 5.5 s for bleaching and coloration, respectively. Kim et al. demonstrated a blue poly(3,3‐bis(bromomethyl)‐3,4‐dihydro‐2H‐thieno[3,4‐b][1,4]dioxepine) (PR‐Br) electrochromic conjugated polymer for an electrochromic capacitive window.^[^ ^25^ ^]^ Through assembling a thin polyaniline (PANI) film as the capacitive layer, the electrochromic capacitive windows show high transparency (>72%) and color contrast (>60%). While such a window displayed a discharging process, it still required the application of an external voltage to fully bleach the device (**Figure** **3**a). Wang et al. further established the energy transferability of the PR–Br‐based electrochromic capacitive windows (Figure 3b),^[^ ^26^ ^]^ with the ability to power a red LED after charged/bleached at 1.8 V. Nonetheless, an external voltage was required to fully color the device.

##### Anodic Electrochromic Materials

**Figure 4:**
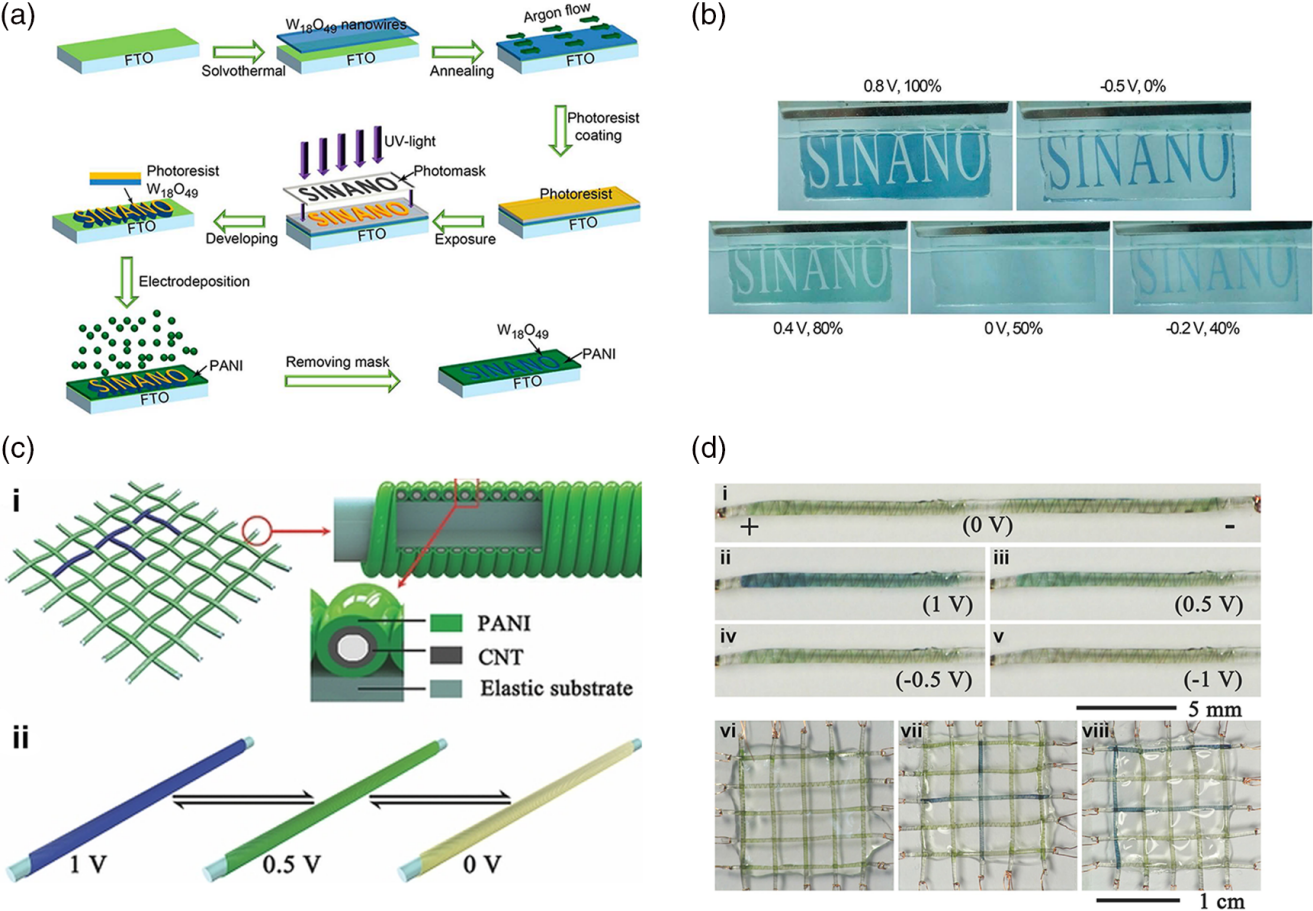
a) Schematic illustration of the fabrication of a patterned W_18_O_49_–PANI composite electrochromic supercapacitor electrode. b) Images of the electrochromic supercapacitor electrode at different energy states, demonstrating the visual energy level indicating functionality. a,b) Reproduced with permission.^[^ ^71^ ^]^ Copyright 2014, American Chemical Society. c) Schematic illustration of the fabrication of an electrochromic fiber‐shaped supercapacitor. d) Images of the electrochromic fiber‐shaped supercapacitors. c,d) Reproduced with permission.^[^ ^72^ ^]^ Copyright 2014, Wiley‐VCH.

The anodic electrochromic materials are another class of electrochromic materials for electrochromic supercapacitor applications. Cai et al. presented NiO nanoparticle film as a promising electrode for electrochromic supercapacitors,^[^ ^68^ ^]^ whereas Chen et al. showed that NiO nanoflake glass electrodes possessed superior cyclic stability.^[^ ^69^ ^]^ The NiO nanoflake electrode changed its color from light brown to black while expressing a light modulation of 40% and a high coloration efficiency of 63.2 cm^2^ C^−1^ at a wavelength of 632.8 nm. MoO_3_ nanobelt/Ni(OH)_2_ nanosheet composite for anodically coloring electrochromic supercapacitors were demonstrated by Zhu et al.^[^ ^70^ ^]^ When the three charged/colored electrochromic supercapacitors were connected in series, they were managed to supply enough voltage to power an LED. Tian et al. utilized anodic coloring PANI for electrochromic supercapacitor electrodes having an energy level indicating functionality.^[^ ^71^ ^]^ Here, the smart electrodes were fabricated via a continuous photolithography and electrodeposition method, which were used for designing patterns (**Figure** **4**a). The patterns changed colors in response to the charging/discharge processes with a light modulation of ≈80% (Figure 4b).

A fiber‐shaped supercapacitor was demonstrated by Chen et al. The electrochromic fiber‐shaped supercapacitor was achieved by depositing PANI onto sheets of aligned carbon nanotubes acting as the electrodes.^[^ ^72^ ^]^ These fiber‐shaped supercapacitors could be further woven into fabrics to display designed patterns (Figure 4c). Figure 4d shows the fiber‐shaped supercapacitor and woven fabrics displaying different colors when supplied with different voltages. The color‐changing can visually demonstrate the energy storage state during the self‐discharge process, which lasts for 70 h. Notably, these chromatic transitions were capable of cycling for more than 3000 cycles without expressing fatigue.

##### Perspectives

It is well‐accepted that electrochromic supercapacitors are unique. They can be used to visualize the energy levels in energy storage devices and recycle a fraction of the consumed energy. Recently, efforts have been devoted to realizing high capacitance; however, the inherent differences between electrochromic devices and capacitors have been greatly overlooked.^[^ ^73^ ^]^ For an electrochromic device, the main objective is to obtain a high optical contrast at low energy consumption. However, the pursuing of high capacitance in supercapacitors is contrary to the inherent low capacitance of thin electrochromic films. As such, a full‐integrated electrochromic supercapacitor must achieve balanced performance between electrochromism and capacitance.

#### 2.2.2 Electrochromic Batteries

Electrochromic material can be incorporated as a battery cathode to express both the electrochromic phenomena and battery function in a single platform. Considering the operating principle of an electrochromic battery (where a metal layer serves as the anode and an electrochromic layer serves as the cathode), the material for electrochromic battery can be classified into two types: metallic anode materials and electrochromic battery materials. The redox potential difference between anode and cathode provides the driving force to oxidize the metallic materials and to reduce the electrochromic materials. Therefore, an electrochromic battery can spontaneously tune the light absorption and provide an excelling function to retrieve the energy consumed during the reverse light modulation process.

##### Metallic Anode Materials

**Figure 5:**
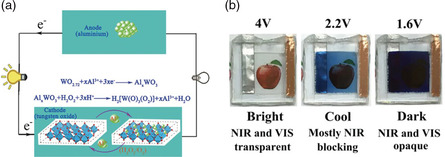
a) Schematic illustration of the working mechanism for H_2_O_2_‐assisted Al–tungsten oxide electrochromic battery. Reproduced with permission.^[^ ^75^ ^]^ Copyright 2016, Wiley‐VCH. b) Digital photos of a prelithiated Al (PLAl) anode‐based electrochromic battery in bright, cool, and dark modes. Reproduced with permission.^[^ ^31^ ^]^ Copyright 2020, Wiley‐VCH.

For an electrochromic battery application, active metals (e.g., Al, Zn) offer significant advantages, such as having high theoretical specific capacity, low cost, and being highly abundant.^[^ 28, 74 ^]^ Zhao et al. introduced an H_2_O_2_‐assisted Al‐tungsten oxide electrochromic battery.^[^ ^75^ ^]^ Here, when tungsten oxide and Al electrodes are connected, Al, being the active metal, easily releases some of its electrons to form Al^3+^ ions; whereas tungsten oxide acts as an oxidant to acquire the released electrons (**Figure** **5**a). The Al–tungsten oxide electrochromic battery was shown to exhibit an open‐circuit potential (OCP) of about 1.2 V, which enables the self‐coloration process of tungsten oxide to take place. However, such an assembled electrochromic battery requires the presence of H_2_O_2_ for the charging process (bleaching of tungsten oxide), which cannot be operated as a closed system. Introducing O_2_ into the electrochromic battery system is another common charging method for an Al‐based electrochromic battery. Wang et al. reported O_2_‐assisted Al‐Prussian blue (PB) electrochromic battery.^[^ ^76^ ^]^ For this PB/Al cell configuration, O_2_ plays a key role in the oxidation process of PB. The addition of a small amount of H_2_O_2_ or O_2_ accelerates the oxidation reaction of the reduced electrochromic layers and consequently decreases the charging time substantially. Furthermore, the “deep” active sites in the electrochromic materials, that are difficult to access, can be activated by H_2_O_2_ or O_2_, and thus, leading to enhanced storage capacity.^[^ 75, 76 ^]^ In general, these Al‐based electrochromic batteries were operated in an open system with a special charging strategy (i.e., introducing H_2_O_2_ or O_2_ to the system). Unfortunately, an open system electrochromic battery is not a versatile platform for most applications. Recently, Zhang et al. introduced a LiAl/Al anode to overcome the operating difficulties of the Al‐based electrochromic battery.^[^ ^31^ ^]^ However, the high operating potential (4 V) leads to high energy consumption during the bleaching process (Figure 5b).

**Figure 6:**
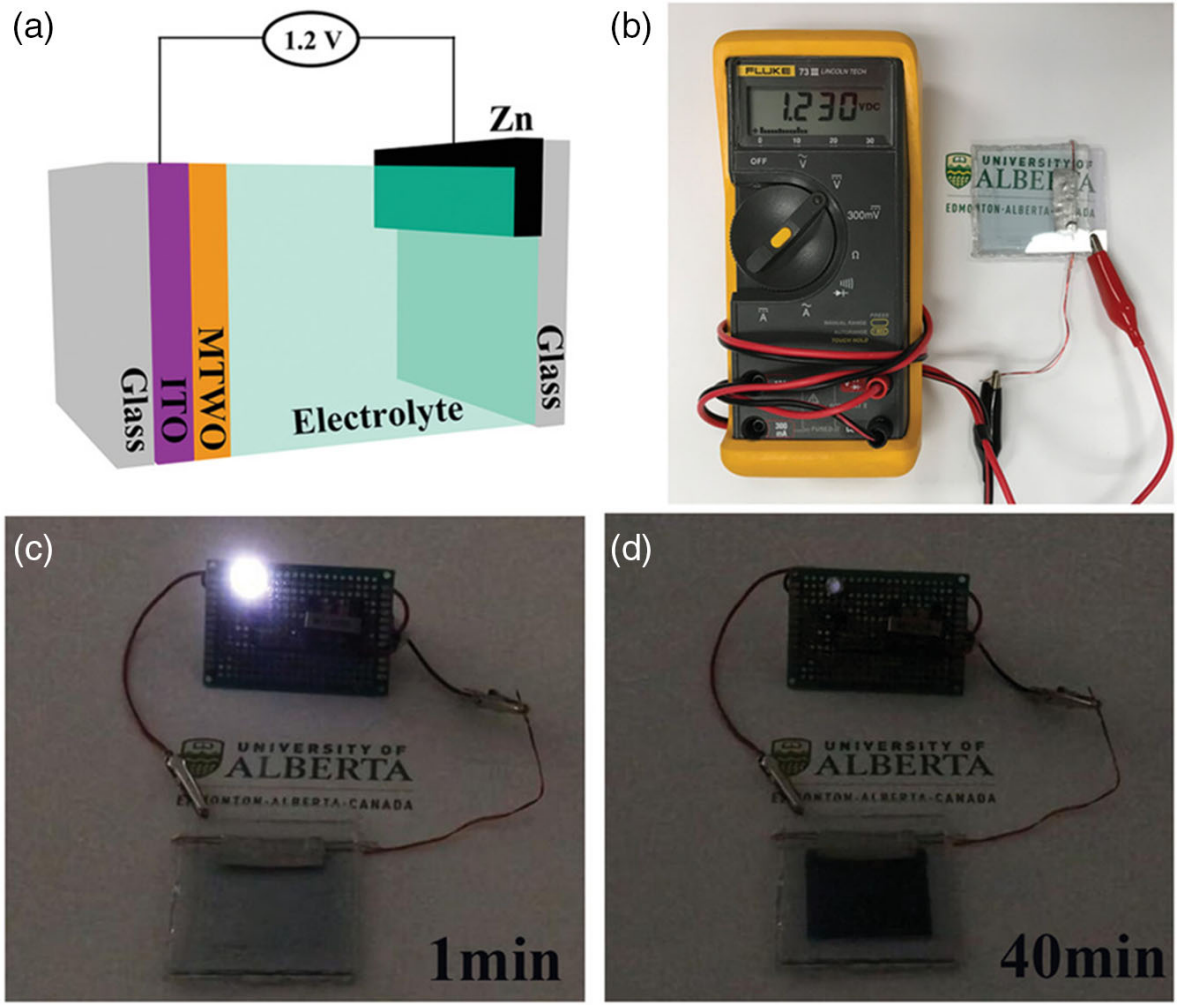
a) Schematic configuration of the Zn‐MTWO electrochromic battery. b–d) Images of a 0.5 V LED powered by the electrochromic battery that possesses 1.23 V OCP. a–d) Reproduced with permission.^[^ ^28^ ^]^ Copyright 2019, Wiley‐VCH.

In 2019, we developed a promising Zn‐based electrochromic battery technology.^[^ ^28^ ^]^ Compared with the Li‐ and Al‐based electrochromic batteries,^[^ 31, 32, 33 ^]^ the aqueous compatible Zn anode bears a much lower operating potentials for a Zn‐based electrochromic battery. With a charging potential of 1.2 V, the energy consumption during the bleaching process is low. As such, the Zn‐based aqueous electrochromic battery platform is a true energy‐efficient electrochromic technology. A Zn‐MTWO (Ti‐substituted tungsten molybdenum oxide) electrochromic battery can be fabricated via sandwiching a piece of Zn foil between a spray‐coated MTWO electrode and a bare glass substrate (**Figure** **6**a). The spray‐coated MTWO cathode, triggered by Zn^2+^ intercalation, can be easily recharged by an external voltage and can function in a closed system having an aqueous electrolyte (i.e., due to the lower redox potential of Zn^2+^/Zn). We demonstrated that an assembled Zn‐MTWO electrochromic battery possesses an OCP of 1.23 V (Figure 6b) in a fully charged state, which enables a self‐coloration behavior and energy retrieval functionality. This built‐in voltage allows the battery to switch its color from transparent to dark blue while powering an LED for more than 40 min (Figure 6c,d). This Zn‐MTWO electrochromic battery exhibited an areal capacity of 150 mAh m^−2^ and a high optical contrast (i.e., 62% at 632.8 nm). Unlike the conventional electrochromic devices which consume energy in both coloration and bleaching processes, the ZECDs consume energy in only one process. In this way, the ZECDs enable partial retrieval of the energy consumed in the bleaching process and this electrical energy can be used to power external electronic devices during the self‐coloration process. Such an energy retrieval functionality drastically reduces the total energy consumption of electrochromic batteries compared with conventional electrochromic energy storage devices.

**Figure 7:**
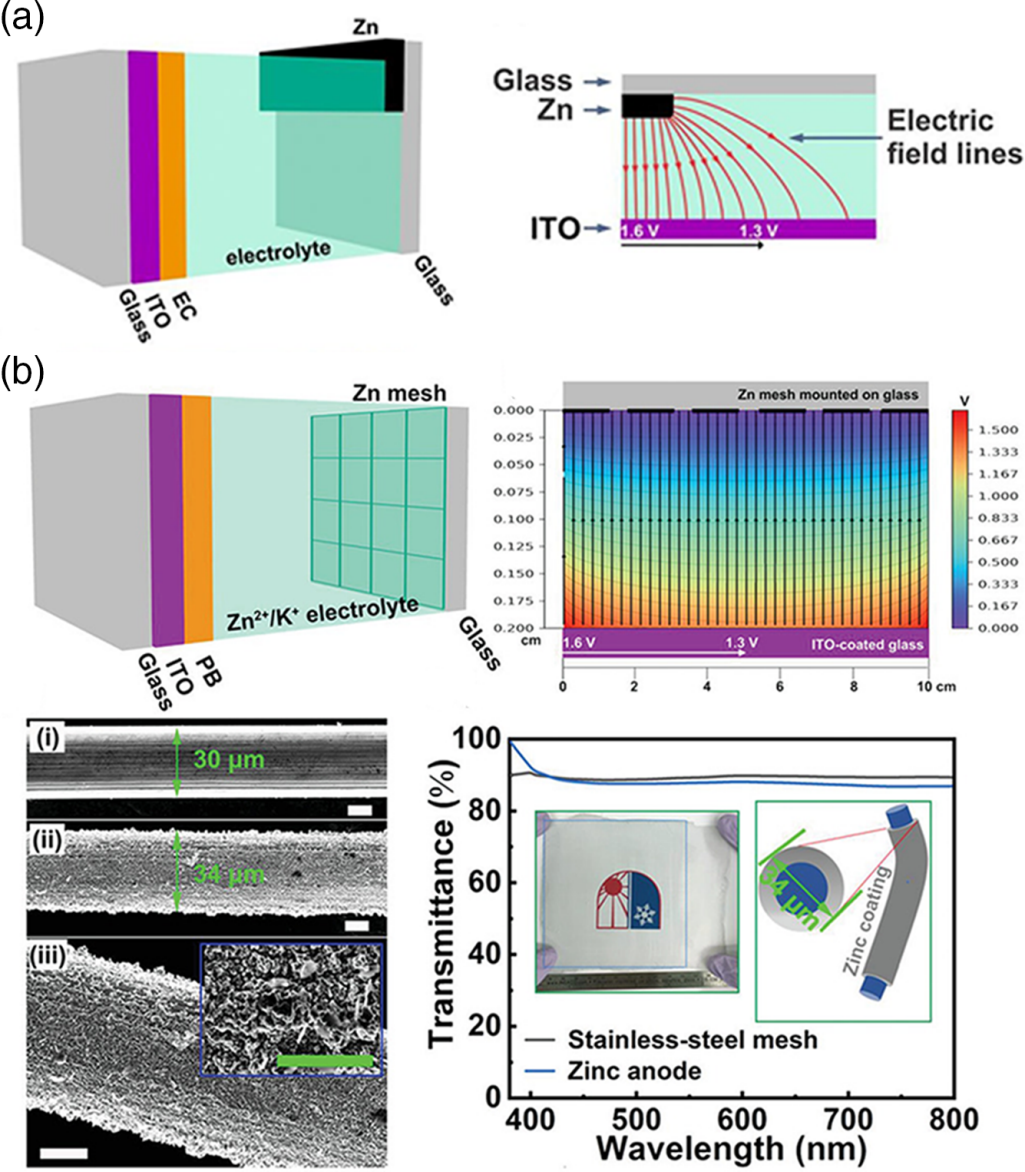
a) Schematic illustration of the nonuniform electrical field lines for a typical zinc foil used in ZECD architecture. b) The architecture and characterization of transparent Zn anode mesh electric field spatial distribution. a,b) Reproduced with permission.^[^ ^30^ ^]^ Copyright 2020, Wiley‐VCH.

In an electrochromic battery, the thick metal films (e.g., Al, Zn) are opaque to light transmission. However, a see‐through electrochromic device is required for transparent battery. Previously, to enable the see‐through ability in ZECDs, a piece of thick flat Zn anode was placed onto a conductive substrate and covered a small fraction of glass (**Figure** **7**a). In this way, light transmission is maintained in the remaining part of devices. However, this arrangement between the thick and opaque flat Zn anode and the electrochromic film cathode produces nonuniform electrical field spatial distribution (Figure 7a). As such, the long lateral pathways for the transportation of the metal cations lead to spatially irregular coloration contrast and slow switching speeds. To overcome this limitation, a flexible transparent Zn‐mesh anode was introduced.^[^ ^30^ ^]^ A Zn‐mesh anode provides a good electric field spatial distribution needed for uniform color switching (Figure 7b). Here, an electrodeposition method was chosen as a means to coat the Zn nanoparticles on the stainless‐steel wire. The optical transmittance of the Zn‐mesh was measured to be 87.8% at 550 nm, which is very close to the optical transmittance of bare stainless‐steel mesh. The incorporation of a transparent Zn‐mesh anode presents a new paradigm in the development of next‐generation transparent batteries.

##### Electrochromic Battery Materials

**Figure 8:**
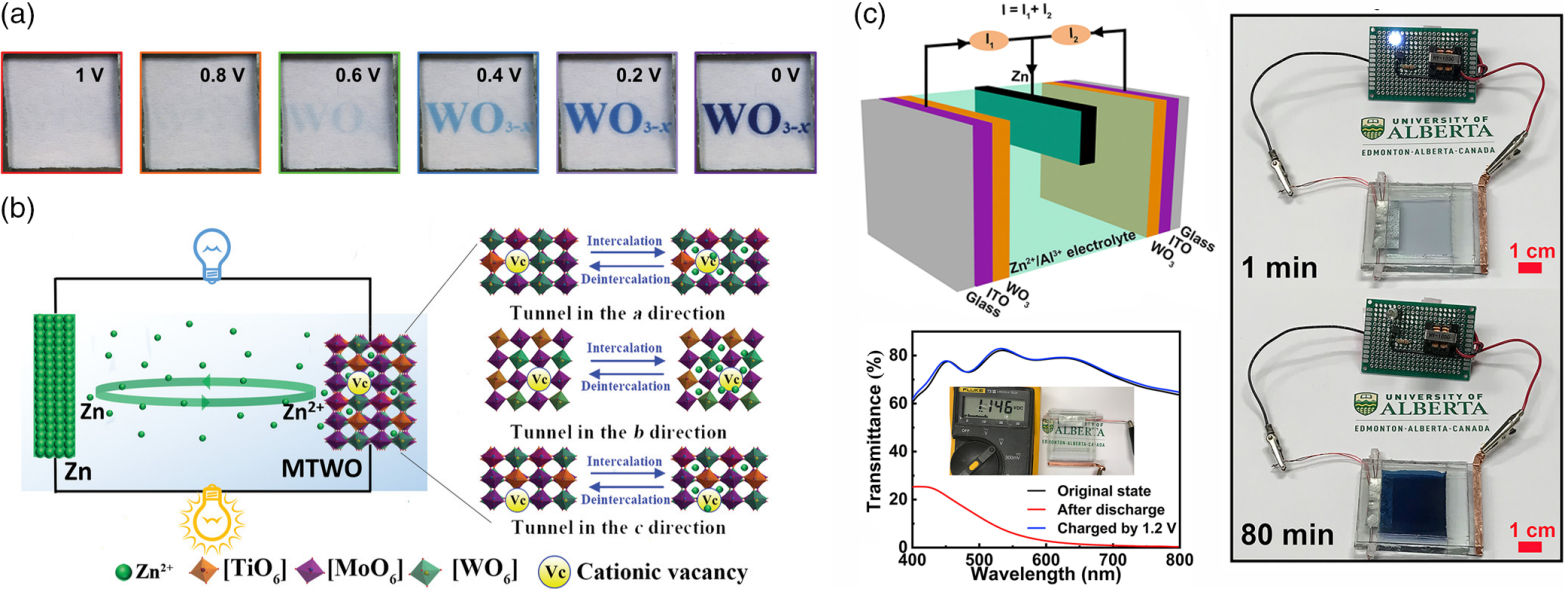
a) Images of Zn‐WO_3–*x*_ electrochromic battery at different states. Reproduced with permission.^[^ ^77^ ^]^ Copyright 2020, Wiley‐VCH. b) Schematic illustration of possible Zn^2+^ intercalation/deintercalation tunnels in MTWO. Reproduced with permission.^[^ ^28^ ^]^ Copyright 2019, Wiley‐VCH. c) Schematic configuration of the Zn^2+^/Al^3+^ electrochromic battery and digital images of a 0.5 V LED powered by the electrochromic battery that possesses 1.146 V OCP. Reproduced with permission.^[^ ^29^ ^]^ Copyright 2019, Elsevier.

Recent investigations of ZECDs electrochromic cathode material have provided various potential applications for transparent batteries. Zhang et al. introduced an additive‐free tungsten oxide nanoparticle for ink‐jet printing of thin films where the WO_3−*x*_ electrode was assembled in a ZECD to display patterns.^[^ ^77^ ^]^ The patterns can switch colors in response to the charging/discharging processes with a high coloration efficiency of 97.7 cm^2^ C^−1^ at 0.4 V and fast responses of 3.7/4.5 s for bleaching/coloration, respectively (**Figure** **8**a). Aqueous MTWO colloid by sequentially exchanging W^6+^/Mo^6+^ with Ti^4+^ via a wet‐chemical doping route was recently synthesized by Li et al.^[^ ^28^ ^]^ The doping process introduces cationic vacancies that act as intercalation sites to unlock the electrochemical activity toward Zn^2+^ ions (Figure 8b). This MTWO cathode exhibited an areal capacity of 260 mAh m^−2^ and high optical contrast of 76%. An alternative ZECD having a Zn anode sandwiched between two electrodeposited WO_3_ cathodes was demonstrated by Li et al. in 1 m ZnSO_4_–AlCl_3_ electrolyte (Figure 8c).^[^ ^29^ ^]^ Compared with the conventional electrochromic devices, these ZECDs were configured by sandwiching a zinc anode between two electrochromic electrodes. In this way, the zinc anode works as both the source of Zn^2+^ ions for coloration and the place of plating Zn^2+^ ions for bleaching. Hence, the ion storage film of the conventional electrochromic devices can be replaced with another electrochromic WO_3_ layer in ZECDs. The optical transmittance spectra of the electrochromic battery are shown in Figure 8c, where the as‐assembled device exhibits high transparency (≈79% at 632.8 nm) and an OCP of 1.146 V. This OCP enables powering of a 0.5 V LED for ≈80 min until the depletion of the ZECD battery. Due to the effect of dual electrochromic layers, this Zn^2+^/Al^3+^ electrochromic device exhibited the highest optical contrast (79%) compared with the current state‐of‐art electrochromic devices.^[^ ^29^ ^]^

**Figure 9:**
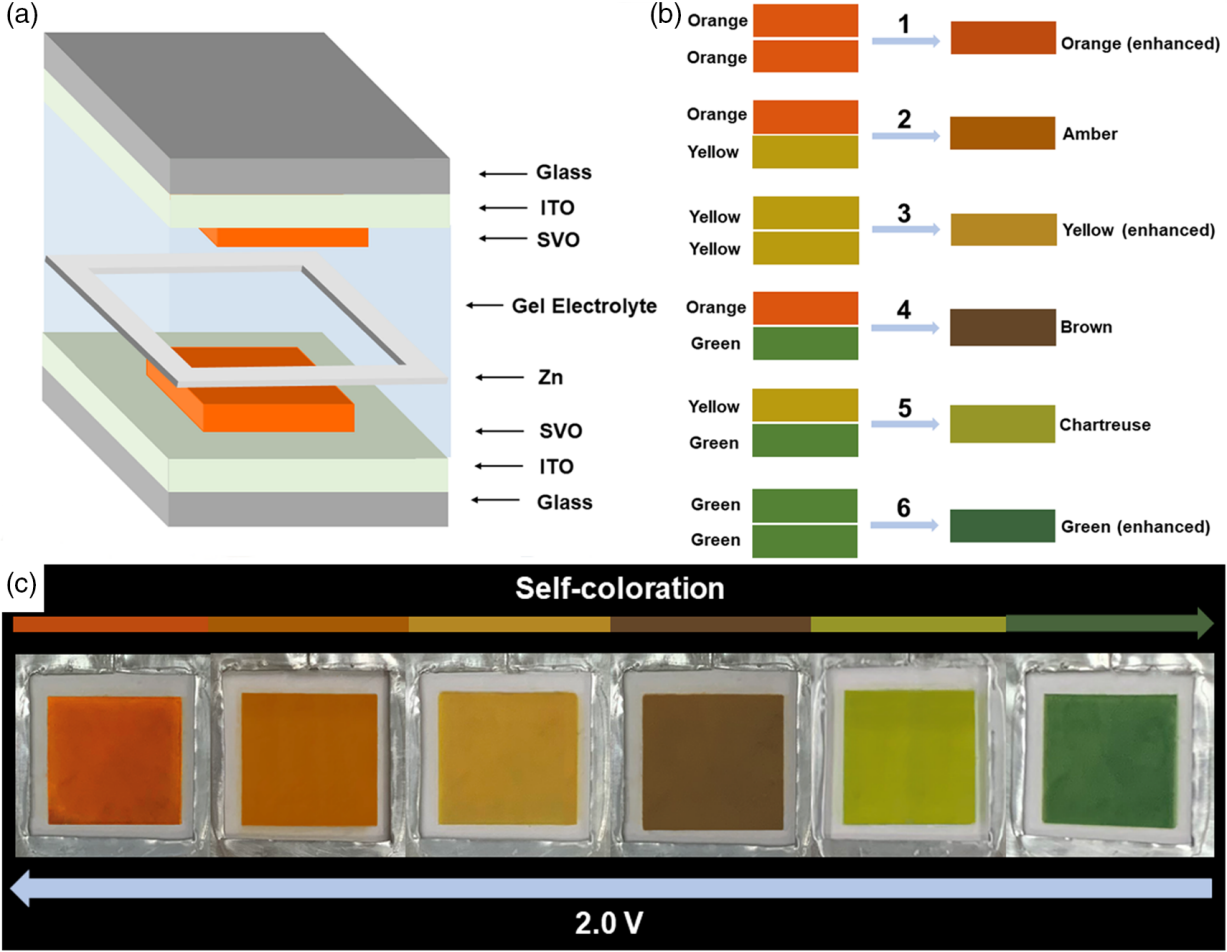
a) Schematic illustration of the Zn–SVO electrochromic display. b) Schematic illustration of the color overlay effect via the combination of orange, yellow, and green colors. c) Digital photographs of the Zn–SVO display showing six colors obtained through the color overlay effect. a–c) Reproduced with permission.^[^ ^78^ ^]^ Copyright 2020, Nature Publishing Group.

The dual electrochromic layers can be further applied in the transparent electrochromic displays.^[^ 78, 79 ^]^ Notably, as the zinc anode is sandwiched between the two electrochromic electrodes, the ZECDs enables independent coloration of top and bottom electrochromic electrodes independently. Such a platform provides an additional degree of flexibility through the utilization of dual electrochromic layers which can be configured to be under the same or at different color states. In this way, the color overlay effect can greatly broaden the addressed color palettes. One such example utilizes NaV_3_O_8_·1.5H_2_O (SVO) nanorods as an electrochromic cathode material.^[^ ^78^ ^]^ The bar‐coated SVO film exhibited a reversible multicolor switch (orange ⇄ yellow ⇄ green) during Zn^2+^ insertion and extraction processes while achieving a 21% optical contrast at 632.8 nm. A Zn–SVO electrochromic battery was assembled via sandwiching a Zn anode between two SVO electrodes, and a polyvinyl alcohol‐ZnSO_4_ gel was used as the electrolyte (**Figure** **9**a). Through the combination of two SVO electrode segments, the color overlay effect broadened the resultant color palettes of the Zn‐SVO display. Figure 9b shows the color overlay effect obtained by superimposing the orange, yellow, and green colors. As the two SVO electrode segments can be colored and bleached independently, the Zn‐SVO device can display six colors (i.e., orange, amber, yellow, brown, chartreuse, and green). This Zn–SVO electrochromic battery possessed an OCP of 1.56 V, which enables the display to spontaneously switch its color from orange to green (including the four intermediate colors). Conversely, the green‐colored display can be recovered to the orange color via a charging process of 2 V (Figure 9c). The dynamic transmittance characteristics of the Zn–SVO electrochromic display were 23.2 s for coloration and 34.8 s for bleaching in a 0.2–2.0 V range. As a metal anode is sandwiched between two electrochromic electrodes, this color overlay effect can be further applied to an electrochromic battery having two different electrochromic cathode materials. Wang et al. reported an electrochromic battery that was assembled based on WO_3_ and Ti‐V_2_O_5_ electrochromic cathodes.^[^ ^80^ ^]^ Six different color states were displayed using such a device. The as‐assembled electrochromic battery provided an OCP up to 3.5 V with an areal capacitance of 933 mAh m^−2^.

**Figure 10:**
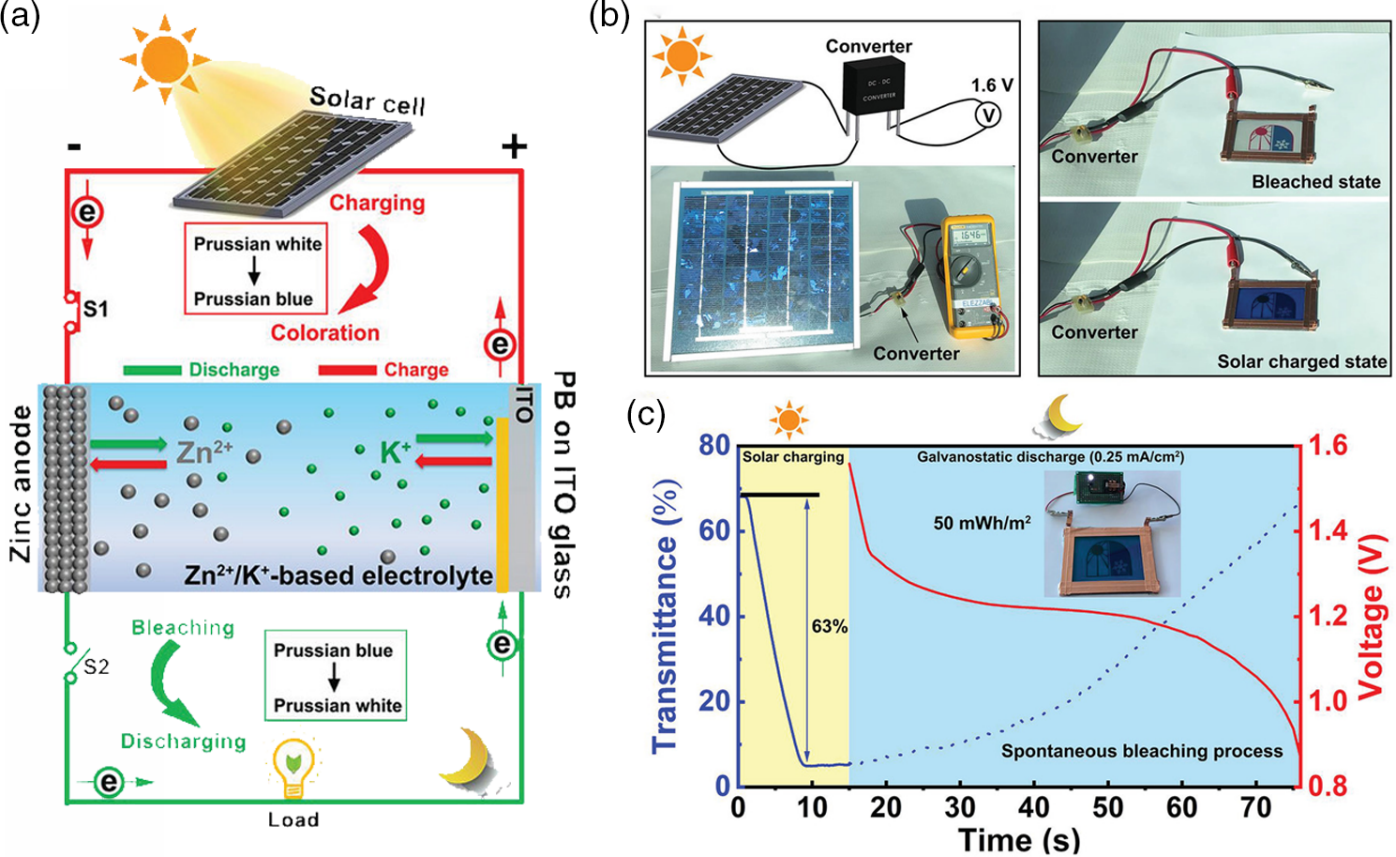
a) Schematic of the PV‐ZECD smart window architecture and the color switching process. b) Photographs of the 80 cm^2^ PV‐ZECD smart window in its colored and bleached states as it is charged by the PV solar panel and when discharged. c) Optical transmission at a wavelength of 632.8 nm during the solar‐charging process (solid blue line), and the discharge process at a current density of 0.25 mA m^−2^ (dotted blue line). The red line is the corresponding galvanostatic discharge curve, and the inset image shows an LED (0.5 V regulated) being lit up by the solar‐charged ZECD smart window. a–c) Reproduced with permission.^[^ ^30^ ^]^ Copyright 2020, Wiley‐VCH.

Anodic electrochromic materials (e.g., PB) have also been utilized as electrochromic battery materials.^[^ 81, 82 ^]^ Anodic electrochromic materials tint under ion extraction such that the electrochromic battery having anodic electrochromic material bleaches during a discharging process (ion intercalation). Hence, ZECDs using anodic electrochromic material as the electrochromic layer is perfectly suited for solar‐charging smart windows since it inherently addresses the solar intermittency issue.^[^ ^30^ ^]^ The working principle of the solar‐charging smart window system is shown in **Figure** **10**a, where PB is being utilized as the electrochromic layer. During daytime, the photovoltaic (PV) solar cell converts sunlight energy and supplies the necessary electrical power required to charge and induce a coloration effect in the Zn–PB electrochromic device. At night or during sunlight intermittency, the colored electrochromic device can be spontaneously bleached while powering an external electrical load (e.g., an LED). As such, the PV‐ZECD smart window system does not require an external power supply at night or during sunlight intermittency conditions. The overall smart window platform provides an efficient strategy to regulate the sunlight on demand. Compared with this PV‐ZECD smart window, the requirement of an external energy source in the conventional electrochromic devices brings a few drawbacks, including complicated installation, increased cost, and offsetting energy savings.^[^ ^83^ ^]^ Figure 10b shows an image of a PV–ZECD smart window. The bleached ZECD smart window can be colored by the voltage supplied from a silicon PV solar panel. Figure 10c illustrates the optical transmission of the smart windows during the solar‐charging process (solid blue line) and the discharge process at a current density of 0.25 mA m^−2^ (dotted blue line). The energy released from the solar‐charged ZECD window, along with the discharging process at 0.25 mA cm^−2^, was calculated to be 50 mWh m^−2^. The PV‐ZECD smart window can be colored within 10 s and bleached within 1 min, accompanied by a high optical contrast (63%). The inset of Figure 10c shows that the colored electrochromic part can power an 0.5 V regulated LED during the discharging process.

##### Perspectives

A battery device requires high capacity while an efficient electrochromic phenomenon requires fast switching speed. The active material film in Zn anode‐based electrochromic device is typically very thin to obtain a fast switching speed. This, in turn, reduces the amount of stored energy within the device. Hence, in designing an integrated Zn anode‐based electrochromic device, a proper balance must be struck between the electrochromic performance and the energy storage capacity. Moreover, while the low discharging voltage results in a rapid coloration process, it reduces the amount of electrical energy retrieved from the device. There is an interplay between rapid color switching and high electrical energy retrieval capability which can be potentially surmounted with the introduction of nanostructured and doped electrochromic materials.

### 2.3 Electrolytes

**Figure 11:**
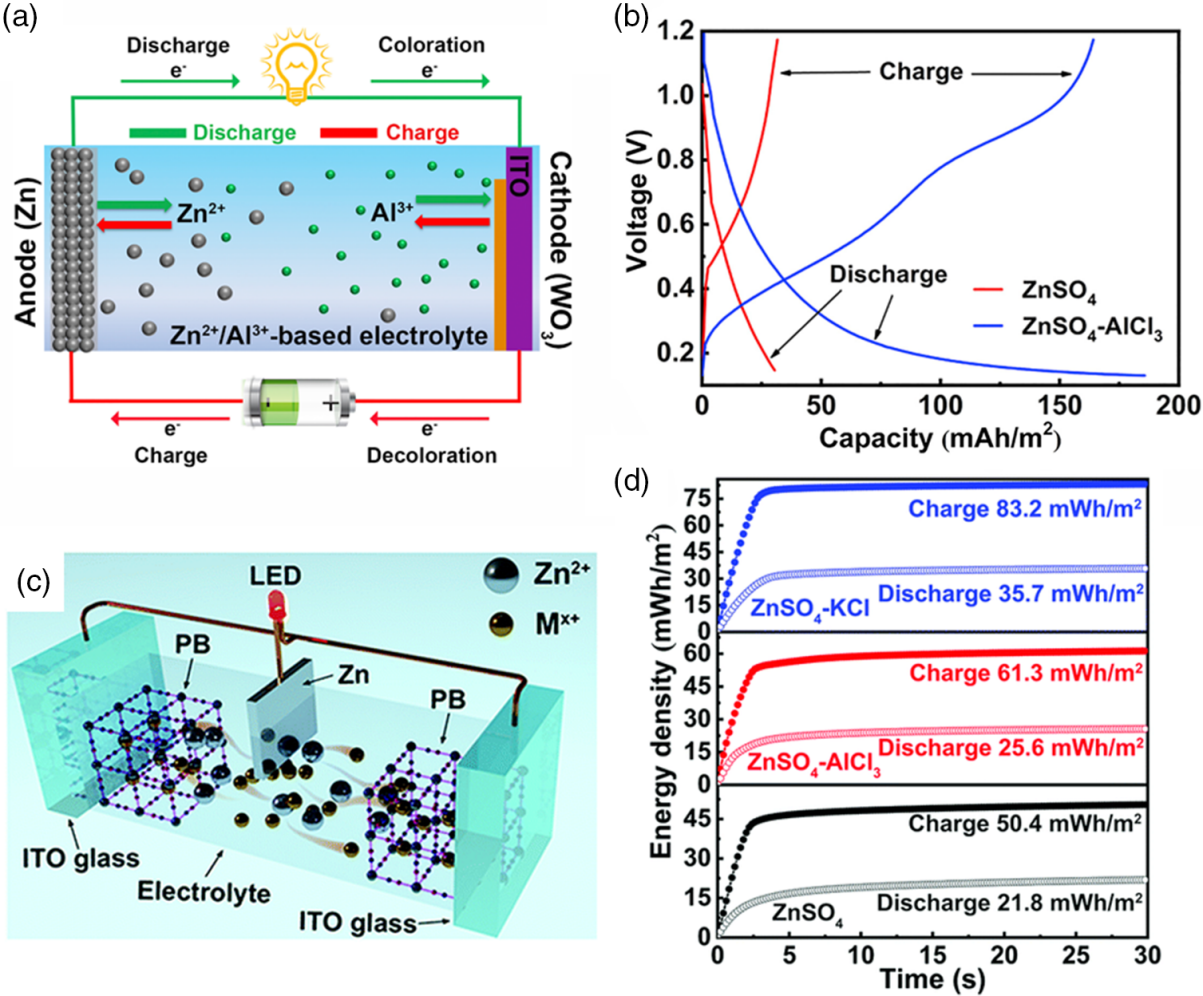
a) Schematic illustration of the rechargeable aqueous hybrid Zn^2+^/Al^3+^ electrochromic battery. b) Galvanostatic charge and discharge curves of the WO_3_ cathode at 0.5 mA cm^−2^ between 0.1 and 1.2 V in different electrolytes. a,b) Reproduced with permission.^[^ ^29^ ^]^ Copyright 2019, Elsevier. c) Schematic illustration of the Zn–PB electrochromic battery. d) Round‐trip energy density comparison of the PB cathodes during dynamic tests in different electrolytes. c,d) Reproduced with permission.^[^ ^81^ ^]^ Copyright 2020, Royal Society of Chemistry.

For the conventional electrochromic devices, the commonly used electrolytes are based on monovalent ions (e.g., H^+^, Li^+^).^[^ 51, 84, 85 ^]^ Recently, multivalent cation Al^3+^ was found to provide multiple charges to accelerate the redox reactions for fast electrochromic switching time of WO_3_.^[^ ^74^ ^]^ However, the incorporation of multivalent Al^3+^ cations in an aqueous electrochromic battery is still facing significant challenges because of the high redox potential (−1.68 V vs standard hydrogen electrode) of Al^3+^/Al.^[^ ^31^ ^]^ In ZECDs, the electrochromic material light absorption is triggered by Zn^2+^. The lower redox potential (−0.76 V vs standard hydrogen electrode) of Zn^2+^/Zn endows that the ZECD platform is more compatible with aqueous electrolyte systems.^[^ 28, 29 ^]^ However, the poor kinetics of Zn^2+^ cation in some electrochromic materials (e.g., WO_3_, PB) limits such a process for viable applications.^[^ ^29^ ^]^ This stems from the fact the high activation energy for interfacial charge‐transfer and poor electrochemical activities.^[^ ^28^ ^]^ These factors significantly limit the capacity, switching speed, and optical contrast of the electrochromic material utilizing Zn^2+^. Therefore, a hybrid Zn^2+^/Al^3+^‐based electrolyte system for fast color switching of electrochromic batteries was developed.^[^ ^29^ ^]^ In this hybrid electrochromic battery, Zn is stripped into the hybrid Zn^2+^/Al^3+^‐based electrolyte during the discharging process, and Al^3+^ is embedded into the WO_3_ cathode, thus triggering the coloration of the cathode. Conversely, during the charging process, Zn^2+^ is plated onto Zn foil and Al^3+^ is extracted from the colored WO_3_ cathode, leading to the bleaching of the cathode (**Figure** **11**a). The hybrid Zn^2+^/Al^3+^‐based electrolyte presents a high discharging capacity which is more than six times to the discharging capacity achieved in pure Zn^2+^‐based electrolyte (Figure 11b). A hybrid K^+^/Zn^2+^‐based electrolyte in the Zn–PB electrochromic battery configuration was also investigated (Figure 11c) and showed high energy capacity (i.e., high optical contrast) of PB cathode (Figure 11d). Compared with monovalent K^+^, the multivalent cations (Zn^2+^, Al^3+^) may lead to a large lattice distortion due to the strong electrostatic interactions between embedded cations and PB films.^[^ ^81^ ^]^ Thus, K^+^ is more efficient to trigger the light modulation of PB films. This result is consistent with the consequence reported by Wang et al., where a Zn‐PB electrochromic battery was assembled with hybrid K^+^/Zn^2+^‐based electrolyte.^[^ ^82^ ^]^ Thus, the hybrid electrolyte systems represent a promising strategy to achieve high‐performance ZECDs.

## 3 Conclusion

This Review summarizes the recent progress of ZECDs. The comprehensive comparison of conventional electrochromic devices and ZECDs is presented considering the basic principles, device designs, electrochromic materials, and electrolytes. Although the field of electrochromic batteries is still in its infancy, the self‐coloration behavior and energy retrieval functionality from ZECDs will render highly energy‐efficient devices, especially for large‐area integration. The color overlay effect certainly offers a new coloring paradigm as it broadens the color palettes of electrochromic displays. Furthermore, it is envisioned that the novel PV‐ZECD smart window system will provide a launching point for next‐generation energy‐efficient smart windows. Future research of ZECDs should be more focused on the assembly of high‐performance electrochromic devices for practical use. Notwithstanding, like any newly emerging technology, there are still some challenges that hinder the development of high‐performance ZECDs for real‐world applications. Examples of such challenges include: 1) the energy retrieval functionality is currently poorly understood. A better understanding of this intriguing process will assist in designing more energy‐efficient ZECDs that can reclaim back more of the consumed energy. 2) The ion intercalation mechanism is still not clear, especially for hybrid electrolyte systems and further investigation is needed to optimize such a process. 3) Practical use of ZECDs requires long‐term stability and excellent light modulation. As such, a better understanding for both of the electrochromic material and the electrolytes involved is critical to promote performance and to enhance overall device stability. 4) Similar to conventional electrochromic devices, the ZECDs also suffer from dendrite growth of anodes that degrade the device's performance.

Although such drawbacks need to be remedied, the extraordinary functionalities of ZECDs make them very promising for the future development of electrochromic devices. It is expected that the ZECDs platform will be broadly incorporated in transparent batteries, durable smart windows, multicolor displays, and variable optical devices.

## Conflict of Interest

The authors declare no conflict of interest.
